# Cytological Evaluation of Space-Occupying Lesions of the Liver by Fine Needle Aspiration

**DOI:** 10.7759/cureus.110326

**Published:** 2026-06-05

**Authors:** Sanjeet Kumar, Bipin Kumar, Sanjay Suman, Rajesh K Singh

**Affiliations:** 1 Biochemistry, Employees State Insurance-Post Graduate Institute of Medical Sciences and Research, Employees State Insurance Corporation Medical College and Hospital, Kolkata, IND; 2 Pathology, Indira Gandhi Institute of Medical Sciences, Patna, IND; 3 Radiodiagnosis, Indira Gandhi Institute of Medical Sciences, Patna, IND; 4 Radiation Oncology, Indira Gandhi Institute of Medical Sciences, Patna, IND

**Keywords:** fine needle aspiration cytology (fnac), fnac of liver lesions, hepatocellular carcinoma (hcc), liver sol, space occupying lesions of liver

## Abstract

Introduction

Malignant space-occupying lesions (SOLs) of the liver, particularly hepatocellular carcinoma, are more common in developing countries such as China and India due to cirrhosis, hepatitis B and C, haemochromatosis, and emerging metabolic risk factors. The aim and objective of the study was to evaluate the cytological spectrum and diagnostic yield of USG-guided fine-needle aspiration cytology (FNAC) in SOLs of the liver. Specifically, it focused on differentiating benign from malignant lesions, distinguishing primary hepatic malignancies from secondary (metastatic) tumours, and establishing a cytological diagnosis in cases of SOLs of the liver.

Methods

Fifty-seven patients with a clinical history suggestive of SOLs in the liver on ultrasound imaging, and for whom FNAC was advised by the clinician, were included in the study. Aspirates were taken, and smears were prepared as soon as possible in the laboratory. Air-dried smears were stained with May-Grünwald-Giemsa, while wet-fixed smears were processed for Papanicolaou and haematoxylin-eosin staining. The stained smears were studied for cytomorphology to establish the diagnosis.

Results

During the study period, a total of 57 patients who reported for aspiration of liver SOLs and met the inclusion criteria were included in the study. The mean age at presentation of liver SOLs was 52.2 years, with ages ranging from 24 to 75 years. The most common age group for malignant lesions was 40-60 years, with a male predominance; the male-to-female ratio was 2.4:1. The liver lesions were cytologically classified as non-neoplastic (10 cases, 17.54%), neoplastic (44 cases, 77.19%), and non-diagnostic (3 cases, 5.26%). The commonest malignancy in our study was hepatocellular carcinoma, which accounted for 24 cases (54.54%), followed by metastatic adenocarcinoma in 16 cases (36.36%) and unclassified carcinoma in 4 cases (9.09%).

Conclusion

This study demonstrates that FNAC enables the early and accurate diagnosis of liver SOLs in resource-limited hospitals, thereby reducing associated morbidity and mortality.

## Introduction

Malignant space-occupying lesions (SOLs) of the liver, particularly hepatocellular carcinoma (HCC), are more common in developing countries such as China and India due to cirrhosis, hepatitis B and C infections, haemochromatosis, and emerging metabolic risk factors [1,2]. A space-occupying lesion, by definition, is a discrete abnormality arising within the liver that can be classified as developmental, neoplastic, inflammatory, or miscellaneous [3].

The sonographic appearance of liver metastases can be mimicked by benign lesions, and failure to recognize this possibility may lead to a mistaken diagnosis of carcinoma [4]. USG-guided fine-needle aspiration cytology (FNAC) has now emerged as a very simple, safe, cost-effective, minimally invasive, and rapid modality for the cytological evaluation of liver lesions and has substantially reduced the reliance on conventional large-needle core biopsy in the diagnosis of focal hepatic lesions [5]. Its principal advantage lies in the ability to perform multiple sampling passes, which increase the chance of obtaining adequate viable cells, especially in necrotic tumours [6]. USG-guided FNAC is primarily utilized to distinguish benign from malignant lesions, differentiate primary from secondary malignancies, and establish a definitive diagnosis [5].

The main indications for FNAC of the liver include the presence of single or multiple nodular lesions identified clinically on palpation or detected by USG. The procedure has few contraindications, chiefly haemorrhagic diathesis and the presence of vascular lesions [7]. The aim of this study was to evaluate the cytological spectrum and diagnostic yield of USG-guided FNAC as a first-line investigation for the evaluation of SOLs of the liver and to assess the cytomorphological features useful in differentiating benign from malignant lesions, primary hepatic malignancies from secondary tumours, and in establishing a cytological diagnosis.

## Materials and methods

It was a prospective, descriptive study conducted from June 2014 to August 2014 at a tertiary care super-speciality teaching hospital in Bihar, India. A total of 57 cases were included in the study during the three-month study period.

Inclusion criteria

All patients during study period with clinical history suggestive of SOL in the liver on ultrasound imaging and FNAC advised by clinician were included in the study.

Exclusion criteria 

Patients with haemorrhagic diathesis and/or vascular lesions and patients with an abnormal prothrombin time index were excluded.

Intervention and procedure 

Sample collection and data collection regarding the detailed clinical history and general physical examination, with particular emphasis on the assessment of liver size, were recorded. USG-guided aspirates were taken from the liver lesion using a 22-gauge needle attached to a 10-ml syringe. Rapid on-site evaluation (ROSE) for adequacy assessment was not performed. Aspirates showing grossly visible material in the hub of the needle were considered adequate for smear preparation. Smears were prepared as quickly as possible in the laboratory. Some smears were air-dried for May-Grünwald-Giemsa staining. One smear was wet-fixed in 95% alcohol for Papanicolaou or haematoxylin and eosin staining. The stained smears were studied for cytomorphological evaluation. FNAC smears showing well-preserved cytomorphological features sufficient for diagnosis were categorized as diagnostic, whereas smears with scant cellularity or a haemorrhagic background that were insufficient for definitive interpretation were categorized as non-diagnostic. Diagnostic smears were further evaluated to establish the cytological diagnosis and characterize the nature of the lesion. Cytological interpretation of all FNAC smears was performed by experienced cytopathologists in the Department of Pathology. In cases with diagnostic difficulty, the slides were reviewed jointly by at least two cytopathologists to reach a consensus diagnosis. Interobserver variability was not assessed separately in the present study. Confidentiality of the patients was maintained, and the data obtained were entered and statistically analysed using Microsoft Excel 2013. Continuous variables were expressed as mean and range, while categorical variables were presented as frequency and percentage.

Ethical considerations 

Informed consent was obtained from all participants prior to the procedure, and the study protocol was approved by the Institutional Ethics Committee (IEC), Ref. IGIMS/2014/1050.

## Results

**Figure 1:**
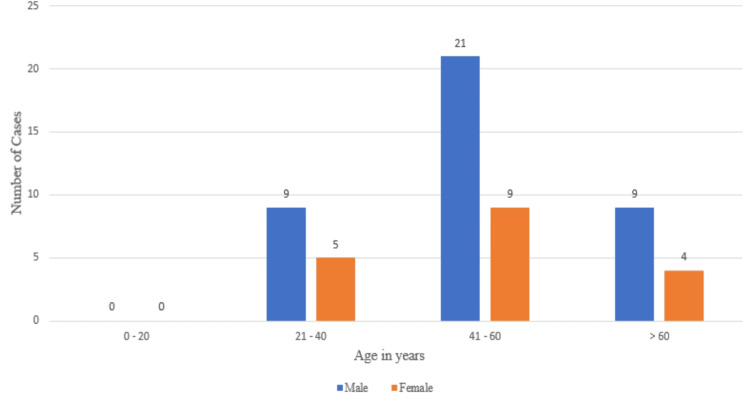
Bar graph showing the age distribution and sex ratio of cases with space-occupying lesions of the liver in different age groups. X-axis: Age in years; Y-axis: Number of cases.

**Figure 2:**
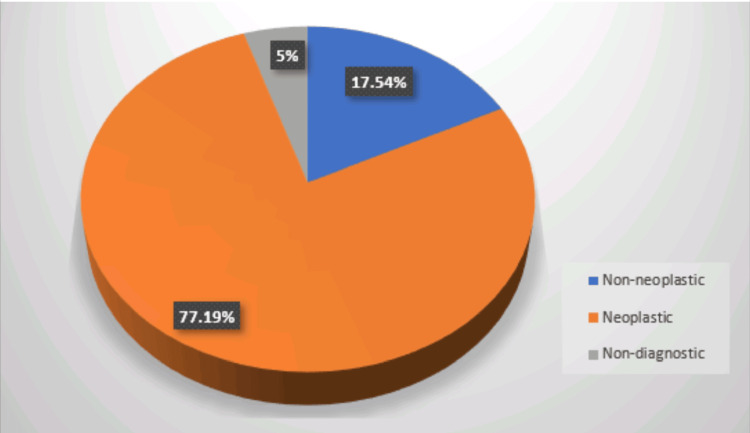
Distribution of different categories of liver lesions.

**Figure 3:**
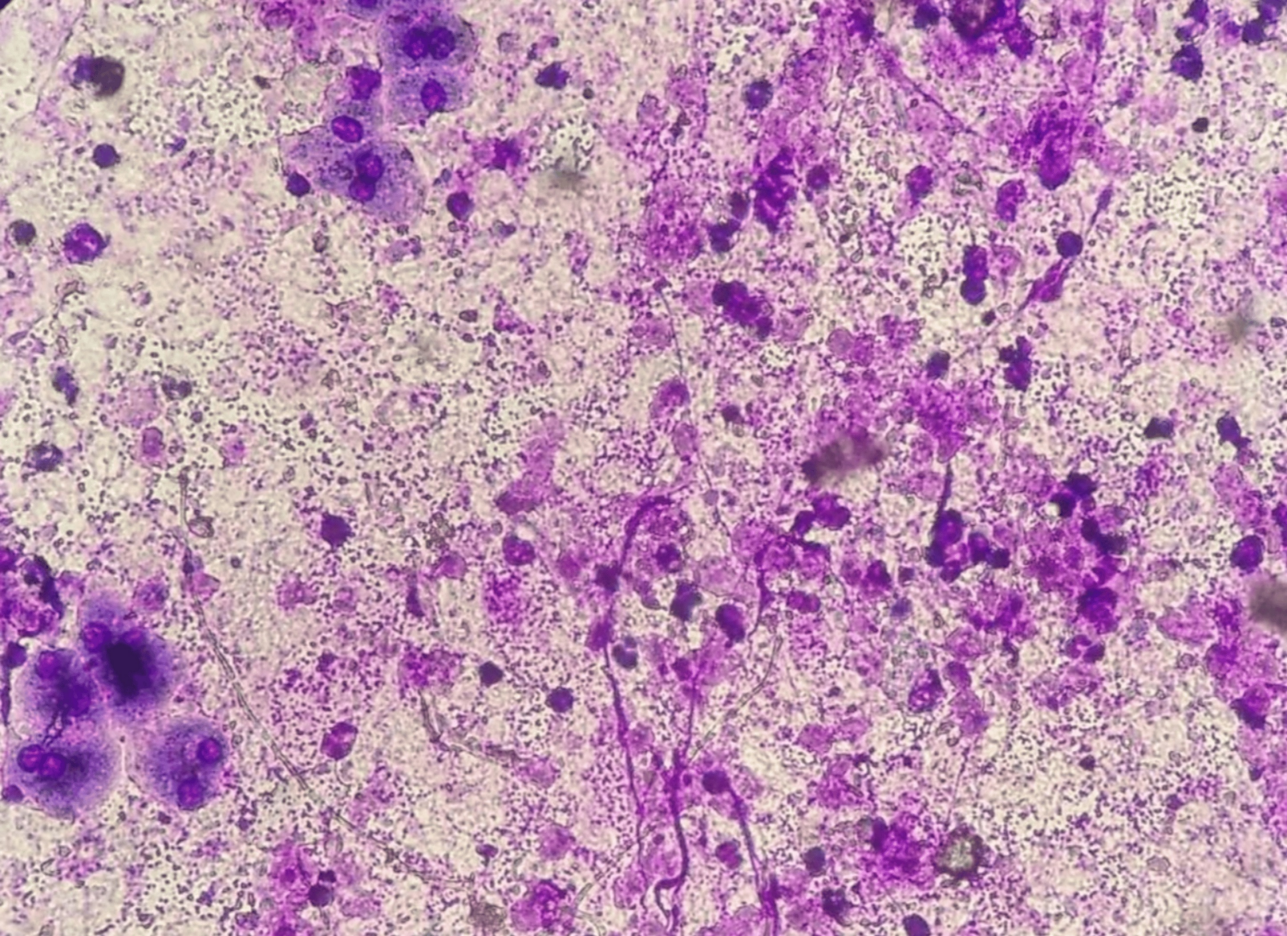
Photomicrograph of a liver abscess showing numerous neutrophils against a necrotic background. Image obtained from a study sample.

**Figure 4:**
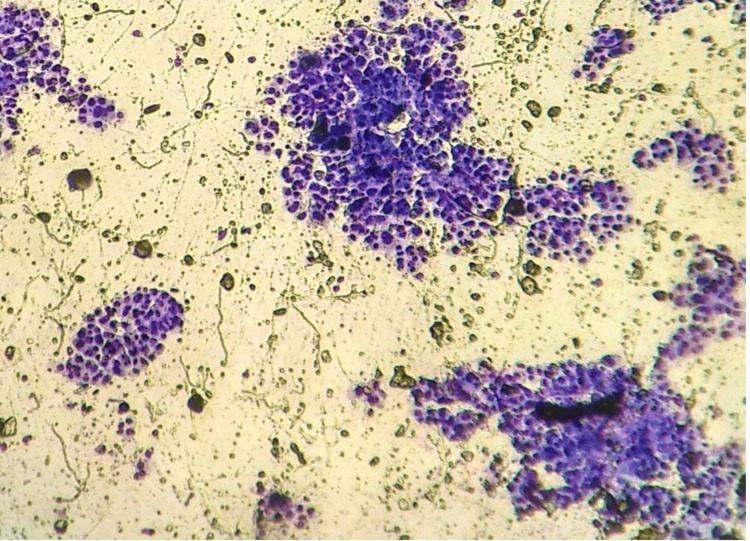
Photomicrograph of hepatocellular carcinoma. Image obtained from a study sample.

**Figure 5:**
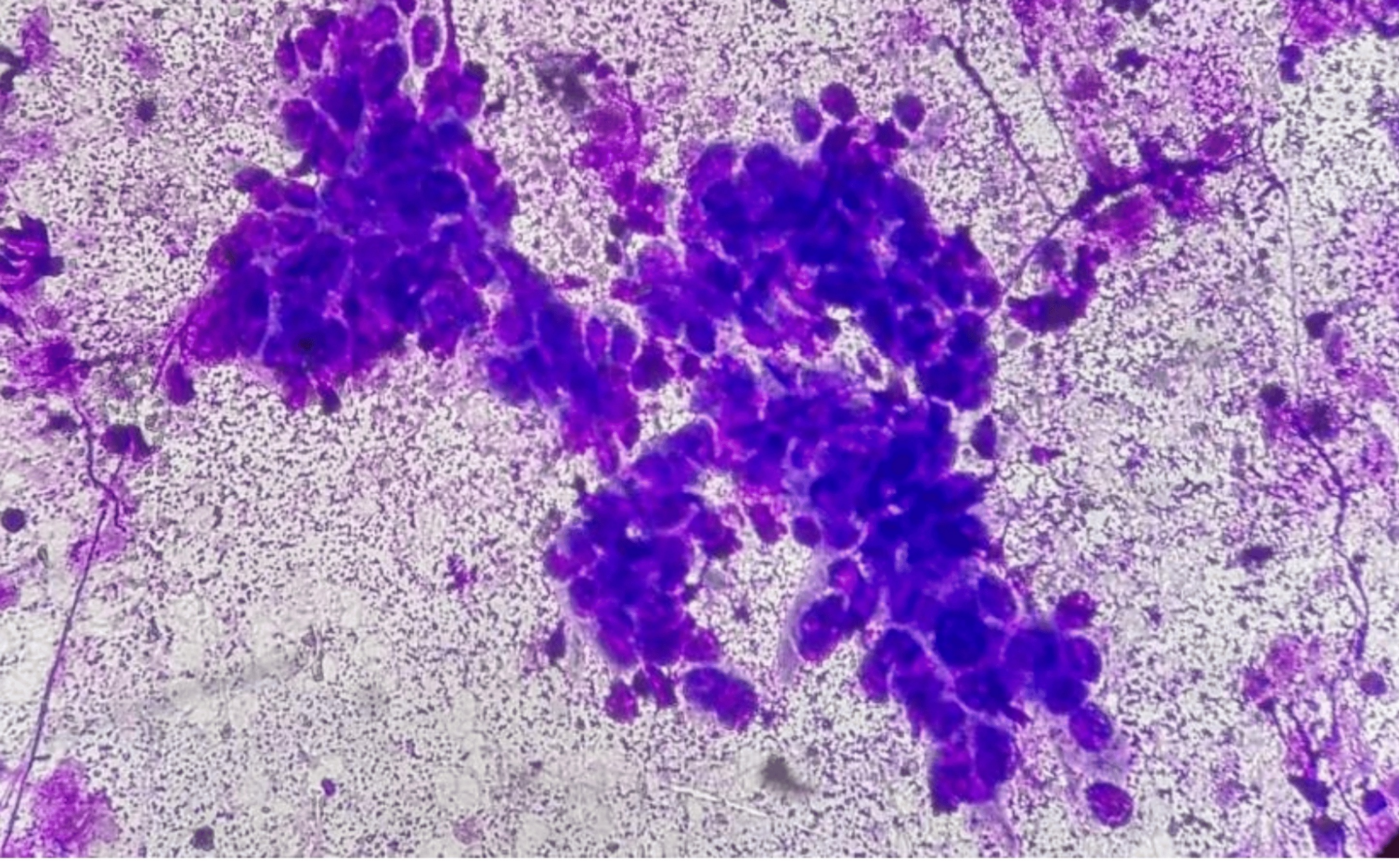
Photomicrograph of metastatic adenocarcinoma. Image obtained from a study sample.

During the study period, consent was obtained from 57 patients who reported for aspiration of liver SOLs and fulfilled the inclusion criteria. The mean age at presentation of liver SOLs was 52.2 years, with ages ranging from 24 to 75 years (Figure 1). For neoplastic lesions, the mean age at presentation was 51.20 years, with patients ranging in age from 24 to 75 years and showing a male predominance; the male-to-female ratio was 2.4:1. The most commonly affected age group for both neoplastic and non-neoplastic lesions was 41-60 years (Table 1). The liver lesions were cytologically classified as non-neoplastic (10 cases, 17.54%), neoplastic (44 cases, 77.19%), and non-diagnostic (3 cases, 5.26%) (Figure 2). Among the non-neoplastic lesions, liver abscesses accounted for 3 cases, granulomatous lesions for 1 case, and regenerating nodules for 6 cases (Table 2). Liver abscess smears (Figure 3) showed predominantly neutrophils against a necrotic background. Granulomatous hepatitis showed clusters of epithelioid histiocytes and multinucleate giant cells, occasionally of the Langhans type. Smears from regenerating nodules showed benign hepatocytes and sheets of benign bile duct epithelial cells. Neoplastic lesions included primary tumours, metastatic adenocarcinoma, and unclassified malignancies (Table 3). The commonest malignancy in our study was HCC, which accounted for 24 cases (54.54%), followed by metastatic adenocarcinoma in 16 cases (36.36%) and unclassified carcinoma in 4 cases (9.09%). The main cytological features of HCC (Figure 4) were hypercellularity with broad trabecular arrangements, endothelial rimming or transgressing vessels within the cell clusters, scattered atypical bare nuclei, a high nucleocytoplasmic ratio, and intranuclear inclusions. The cytological features of metastatic adenocarcinoma were hypercellular smears with predominantly acinar clusters of cells with moderately to markedly pleomorphic, hyperchromatic nuclei (Figure 5). Cases exhibiting unequivocal malignant cytological features but lacking sufficient morphological characteristics to classify them as either primary HCC or a specific metastatic malignancy were categorized as unclassified carcinoma. The commonest clinical presentation was abdominal distension and abdominal pain.

**Table 1:** Distribution of liver lesions (neoplastic, non-neoplastic, and non-diagnostic cases) across different age groups.

Age group (years)	Neoplastic cases	Non-neoplastic cases	Non-diagnostic cases
0-20	0	0	0
21-40	12	0	2
41-60	21	7	1
>60	11	3	0
Total	44	10	3

**Table 2:** Distribution of non-neoplastic cases.

Non-neoplastic cases	No. of cases
Granulomatous lesion	1 (10%)
Regenerating nodules	6 (60%)
Liver abscess	3 (30%)
Total	10

**Table 3:** Distribution of neoplastic cases.

Neoplastic cases	No. of cases
Hepatocellular carcinoma	24 (54.54%)
Metastatic adenocarcinoma	16 (36.36%)
Unclassified carcinoma	4 (9.09%)
Total	44

## Discussion

FNAC, particularly when performed under USG guidance, plays a pivotal role in the evaluation of SOLs of the liver [8]. Although imaging facilitates the early detection of liver abscesses, significant overlap in radiologic features with HCC and metastatic tumours, especially in the presence of necrosis and reactive changes, often makes accurate differentiation challenging [4,9,10]. USG-guided FNAC provides accurate diagnosis with minimal intervention, low cost, and no significant complications [11]. Early diagnosis through image-guided aspiration reduces the need for additional ancillary investigations and shortens the duration of hospital stay [10].

In this study, 77.19% of cases were neoplastic. This high proportion is likely due to the tertiary care setting, where patients often present with complex illnesses and a higher likelihood of referred suspected cancer cases. However, a similarly high proportion of neoplastic cases has been reported by several authors, including Rasania A et al. (67.7%) and Guo Z et al. (66%), supporting the observation that malignant etiologies constitute the majority of aspirated liver lesions in clinical practice [6,12]. The relatively lower proportion of non-neoplastic lesions (17.54%) and non-diagnostic smears (5.26%) in our study further underscores the reliability of FNAC as an initial diagnostic modality for liver lesions.

The mean age at presentation of liver SOLs was 52.2 years, with neoplastic lesions showing a comparable mean age of 51.2 years, which aligns with the established epidemiological trend of liver malignancies occurring in middle to late adulthood [13]. The most common age group for malignant lesions in our study was 40-60 years, with a male predominance; the male-to-female ratio was 2.4:1. This finding aligns with the study conducted by Gatphoh ED et al., in which the commonest age group of malignant liver disease was 51-60 years [14], as well as with global data indicating a higher burden of liver malignancies among males, likely attributable to risk factors such as chronic liver disease, alcohol use, and viral hepatitis [15].

Radiological evaluation of hepatic lesions often presents diagnostic challenges due to overlapping imaging features [9]. There is a degree of overlap in the radiologic characteristics of liver abscess, HCC, and metastatic tumour [16]. Tumours may undergo extensive necrosis, with the resultant radiologic image mimicking abscesses. Multifocal HCC may mimic metastatic tumour radiologically.

FNAC smears showing well-preserved cytomorphological features sufficient to establish a definite cytological diagnosis were categorized as diagnostic, and smears with scant cellularity or a haemorrhagic background that were insufficient for definite interpretation were categorized as non-diagnostic. In our study, FNAC was diagnostic in 94.73% of cases, similar to the study by Goel S et al., which reported that it was diagnostic in 90-95% of cases [8]. Among neoplastic lesions, HCC was the most common malignancy in our study, constituting 54.54% of cases, followed by metastatic adenocarcinoma (36.36%) and unclassified malignancies (9.09%). However, globally, secondary metastatic tumours, most commonly metastatic adenocarcinoma, represent the most frequent malignancies involving the liver, followed by HCC, which is the most common primary hepatic malignancy [17-19]. The possible explanation for the predominance of HCC in our study may be attributed to the high prevalence of chronic liver disease and viral hepatitis in our population [20].

Diagnostic pitfalls in FNAC of liver SOLs include sampling error, scanty cellularity, haemorrhagic smears, and overlapping cytomorphological features, particularly in well-differentiated HCC and poorly differentiated metastatic carcinomas. The cytological criteria helpful in differentiating HCC from metastatic tumours were polygonal cells with centrally placed nuclei, separation of cells by sinusoidal capillaries, and many singly lying atypical bare nuclei [8,21]. Distinctive nuclear changes included intranuclear inclusions and prominent nucleoli. In contrast, metastatic adenocarcinoma exhibited hypercellular smears with predominantly acinar clusters of cells [22] with moderately to markedly pleomorphic, hyperchromatic nuclei.

Clinically, the most common presenting complaints were abdominal distension and abdominal pain, as reported in other studies such as Thyagarajan MS and Sharif K, which are nonspecific but frequently associated with hepatic SOLs [23].

Although chronic infections with hepatitis B and C viruses are predominant risk factors for HCC, other contributors, such as alcohol consumption, tobacco use, and aflatoxin exposure, also play a substantial role, particularly in developing countries [20,24,25]. Furthermore, emerging risk factors, including non-alcoholic fatty liver disease and obesity, are expected to assume increasing significance in the future due to their rising global prevalence [26]. Our study has certain limitations. The study had a small sample size and lacked histopathological correlation. In addition, we did not evaluate the risk factors involved in HCC. Further studies with larger sample sizes and histopathological confirmation are required for better evaluation of diagnostic accuracy and associated risk factors.

## Conclusions

This study highlights the usefulness of FNAC in the diagnosis of SOLs of the liver, particularly in less-equipped clinical laboratories. Radiological imaging modalities such as USG, CT, and MRI have made significant contributions to the detection, localization, and diagnosis of SOLs of the liver, while cytological examination aids in differentiating benign from malignant liver lesions and contributes to early diagnosis. Early diagnosis through USG-guided aspiration may help in timely clinical decision-making and appropriate management of SOLs of the liver.
